# Practical Points

**Published:** 1912-02-24

**Authors:** 


### PRACTICAL POINTS.

(Criticism and Suggestions Invited.)

### III.—How Shorts are Produced.

Damp unfortunately is another very prolific source of shorts. Cables and wires inside all buildings are protected either by wood casing, the wires being laid in grooves in a wooden board, a thin facing-board covering them, or in what are termed steel or iron "conduits." The steel or iron conduits are really pipes. The majority of them are drawn specially for the purpose, but the quality of the conduits varies very considerably, again according to the price that is paid. Sometimes the pipe consists merely of a sheet of the metal rolled round a mandrel, the edges being brought close together. This is the very cheapest form of tube. In other cases a very thin tube is formed in the same way, but the edges are brazed or soldered together. In other forms of tube the pipe is drawn in a proper manner, each length being without longitudinal joint.

Both the wood casing and the conduits are liable to hold moisture when it is present. Wood is very porous, and consequently if it be fixed upon a damp wall, upon a wall exposed to the south-west winds, it is very liable to absorb moisture from the wall like a sponge, and as the wires and cables are more or less clasped by the grooves in which they lie, the moisture is delivered directly on to the insulating envelope or to the lead protecting tube where paper-covered cables are used. With conduits the action is different, but the result is very much the same. With the tubes made with open seams, moisture passes into them when they are laid on a damp wall through the seam, and runs down the inside of the pipe. Where the pipes are closed it may happen that the atmosphere at the time when the wires are being laid is very muggy, the humidity is very high; consequently, a considerable amount of the vapour of water is imprisoned in the tubes when they are closed up. The water so trapped runs down to the lowest point, and there attacks the insulating envelope or the lead covering. With conduits also, joints between adjacent length are necessary. In the best forms of conduit the jointing pieces have been very carefully worked out, so that ingress of moisture through a joint should not be easy. With the cheap forms, however, and with careless workmen, there may be openings at the joints, and the same thing happens as with the openings in the seams. In connection also with the conduit system a number of boxes are necessary for connecting cables and wires together, for holding fuses and switches, coiling roses.

It is necessary that the conduit pipes should be connected to the boxes by waterproof joints. Again, with cheap material or careless work in putting them up, the joints may admit water. The conduits are also subject to another serious drawback where moisture penetrates, unless provision is made; they rust, and the rust may attack the insulating envelope or the lead-protecting tube. All of these things usually occur at some point where the wires make a dip, or where they are run to some low point in the building. In many cases the meter and entry switch are at the lowest part of the building, and the wires have to find their way down to them; in other cases wires have to make dips or curves to avoid obstacles, or to supply lamps or motors in special positions. In all such places any water that happens to be in the pipes will settle, rust will commence unless the pipes are protected, and the insulating envelope or the lead pipe will be attacked. In the best forms of conduit again, careful systems have been worked out for protecting the pipes from rust. Special varnish carefully laid on and baked at a high temperature protects the insides and the outsides of the tubes from rust. Incidentally, it may be noted that when a very thin tube rusts, a hole may easily be formed and moisture from outside can then enter. In the early days of iron and steel conduits they were lined with glass. In later forms paper impregnated with waterproof substances has been used for lining them.

The wood casing may be protected from absorbing moisture by thoroughly drying it in a vacuum oven, or by similar means, and impregnating it with some waterproof insulating substance such as shellac varnish. A very fair result for small pieces of work, such as repairs that may have to be made by the hospital staff, may be obtained by thoroughly drying the wood, say by placing it over a boiler if there is one, then painting the grooves with thin shellac varnish, drying it thoroughly, painting it again and so on, until a good skin of the varnish has been formed where the wires are to lay.

### How the Moisture Attacks the Insulation.

In the case of indiarubber-covered wires, if moisture reaches the rubber it attacks the gums of which the rubber is composed, and it is simply a question of time how soon the rubber shall be so deteriorated that it loses its insulating properties and such mechanical strength as it originally possessed. It practically disintegrates, and its resistance to sparking at the times when high pressures are present, as described in a previous article, is lost.

In the case of paper-covered cables with lead-protecting tubes, if the lead is very good and very flexible it will withstand moisture for a considerable time. If there are any impurities in the lead, however, the moisture will attack them, holes will be formed in the lead, and the insulation of the paper will be destroyed in the manner previously described. Where a lead-covered wire lies in a steel conduit and moisture is present, galvanic action may be set up between the lead and the steel. The insulating varnish on the inside of the steel tube will lessen the action, and may even prevent it, as long as it is intact; but any flaws in the varnish, any spots not properly covered, may give rise to galvanic action at that point, and in time may cause short circuits.

